# Genome Sequence of the Type Strain Azospira restricta SUA2 (DSM 18626)

**DOI:** 10.1128/MRA.00156-21

**Published:** 2021-05-06

**Authors:** Madison C. Mikes, William M. Moe

**Affiliations:** a Department of Civil and Environmental Engineering, Louisiana State University, Baton Rouge, Louisiana, USA; University of Maryland School of Medicine

## Abstract

Azospira restricta SUA2^T^ (DSM 18626) is a Gram-negative-staining bacterium able to fix nitrogen and accumulate polyhydroxybutyrate storage granules. Here, we report the complete genome sequence (3,975,213 bp with 68.64 mol% G+C content), which may prove useful in future efforts to assess the role of *Azospira* in nutrient cycling.

## ANNOUNCEMENT

Azospira restricta SUA2 (=DSM 18626=NRRL B-41660=LMG 23819), the type strain of the species, was isolated from groundwater near Baton Rouge, LA (latitude, 30.581593; longitude, −91.242153), and was demonstrated to fix nitrogen and accumulate polyhydroxybutyrate storage granules (1). Related bacteria ascribed to the genus *Azospira* based on 16S rRNA gene sequencing have been reported in a number of water or wastewater treatment systems employing biological nitrogen or phosphorus removal (2, 3). Few genome sequences are available at present, however, to allow assessment of the genetic basis for the potential roles of *Azospira* spp. in nutrient removal (4, 5).

Azospira restricta SUA2^T^ (=DSM 18626) was obtained from the Deutsche Sammlung von Mikroorganismen und Zellkulturen (Braunschweig, Germany). Cells grown aerobically in Reasoner’s 2A (R2A) broth for 3 days at 37°C were harvested by centrifugation (3,000 × *g*, 4°C, and 30 min), followed by DNA extraction using a GenElute bacterial genomic DNA kit (Sigma-Aldrich). The genome was sequenced using the PacBio Sequel II system (Menlo Park, CA) with v2 chemistry at the Georgia Genomics and Bioinformatics Core (University of Georgia, Athens, GA). Genomic DNA was fragmented to approximately 10 kbp using g-TUBEs (Covaris, Inc., Woburn, MA) and concentrated with bead cleanup prior to SMRTbell library construction according to the procedure for preparing multiplexed microbial SMRTbell libraries for the PacBio Sequel II system. The library including *A. restricta* SUA2^T^ was loaded onto a single-molecule real-time (SMRT) cell according to the diffusion loading protocol. After data demultiplexing, we obtained 266,741 reads (mean subread length, 10,726 bases), covering a total of 13,286,950,038 bases. The genome assembly was performed using SMRT Link v9.0 (Pacific Biosciences) with the input genome size set to 3.5 Mbp. Annotation was performed using the NCBI Prokaryotic Genome Annotation Pipeline v4.13 (6).

The completed genome sequence of *A. restricta* SUA2^T^ comprises a single, circular chromosome of 3,975,213 bp (coverage, 3,100×), with 68.64 mol% G+C content. There are 3,789 predicted genes, including 51 tRNA genes (including those for all 20 standard amino acids plus selenocysteine) and 2 each of 5S, 16S, and 23S rRNA genes.

Consistent with the reported nitrogen-fixing ability of strain SUA2^T^ (1), the genome contains numerous genes annotated as encoding proteins associated with nitrogen fixation. Although it was previously reported that strain SUA2^T^ did not utilize nitrate in an anoxic medium supplemented with lactate (1), the genome contains several genes associated with the reduction of oxidized nitrogen species, including nitrate, nitrite, nitric oxide, and nitrous oxide. In addition to the genes associated with nitrogen cycling, the genome of *A. restricta* SUA2^T^ also contains genes annotated as encoding type I and type II polyphosphate kinases that are associated with polyphosphate accumulation in “*Candidatus* Accumulibacter phosphatis” (7, 8) and *Fluviibacter phosphoraccumulans* (9), which like *Azospira* belong to the order *Rhodocyclales* of the class *Betaproteobacteria*. *A. restricta* may play a larger role than previously known in nutrient cycling.

### Data availability.

The sample information and sequence and genomic assembly/annotation are accessible under the NCBI BioProject, BioSample, and whole-genome sequence accession numbers PRJNA676164, SAMN16756644, and CP064781, respectively. The raw sequencing results are accessible under SRA accession number SRX10051741.
